# Distribution of Biominerals and Mineral-Organic Composites in Plant Trichomes

**DOI:** 10.3389/fbioe.2021.763690

**Published:** 2021-11-19

**Authors:** Hans-Jürgen Ensikat, Maximilian Weigend

**Affiliations:** University of Bonn, Bonn, Germany

**Keywords:** biomineralization, calcium carbonate, calcium phosphate, cell walls, loasaceae, Raman spectroscopy, scanning electron microscopy, trichomes

## Abstract

Biomineralization is a common phenomenon in plants and has been shown to be chemically, functionally and topologically diverse. Silica and calcium carbonate have long been known as structural plant biominerals and calcium phosphate (apatite)–long known from animals–has recently been reported. Strikingly, up to three different biominerals may occur in a single trichome in, e.g., Urticaceae and Loasaceae, and in combination with organic compounds, can form organic/inorganic composite materials. This article presents an extension of previous studies on the distribution of these biominerals in Loasaceae trichomes with a focus on their spatial (three-dimensional) distribution and co-localization with organic substances. Light microscopy and scanning electron microscopy with high-resolution EDX element analyses of sample surfaces and sections illustrate the differential distribution and composition of the different biomineral phases across cell surfaces and cell walls. Raman spectroscopy additionally permits the identification of organic and inorganic compounds side by side. All three biominerals may be found in a nearly pure inorganic phase, e.g., on the plant surfaces and in the barbs of the glochidiate trichomes, or in combination with a larger proportion of organic compounds (cellulose, pectin). The cell lumen may be additionally filled with amorphous mineral deposits. Water-solubility of the mineral fractions differs considerably. Plant trichomes provide an exciting model system for biomineralization and enable the *in-vivo* study of the formation of complex composite materials with different biomineral and organic compounds involved.

## Introduction

**FIGURE 1:**
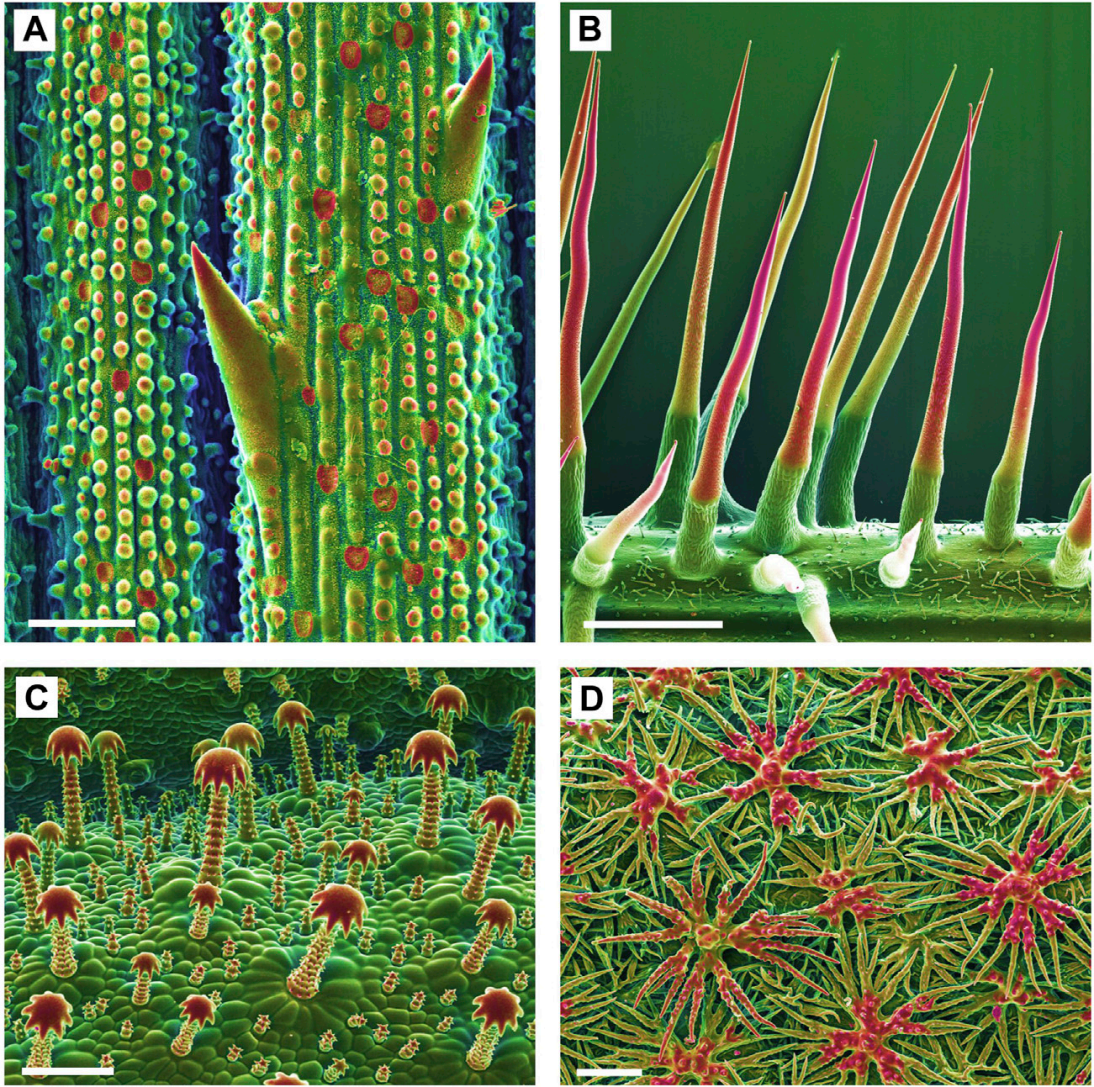
Biomineralized plant surface structures. Combined topographic (SE) and compositional (BSE) contrast SEM images showing mineralized structures in red color. **(A)**: Sharp tips and abrasive epidermis inclusions in a grass (*Spartina pectinata*). **(B)**: Stinging hairs of stinging nettles (*Urtica mairei*). **(C)**: Complex glochidiate trichomes of *Blumenbachia insignis*. **(D)**: A dense cover of the leaf surface with stiff peltate, branched trichomes on Brassicaceae (*Phyllolepidum cyclocarpum*). Scale bars: A = 50 μm; B = 1 mm; C, D = 100 µm.

**FIGURE 2:**
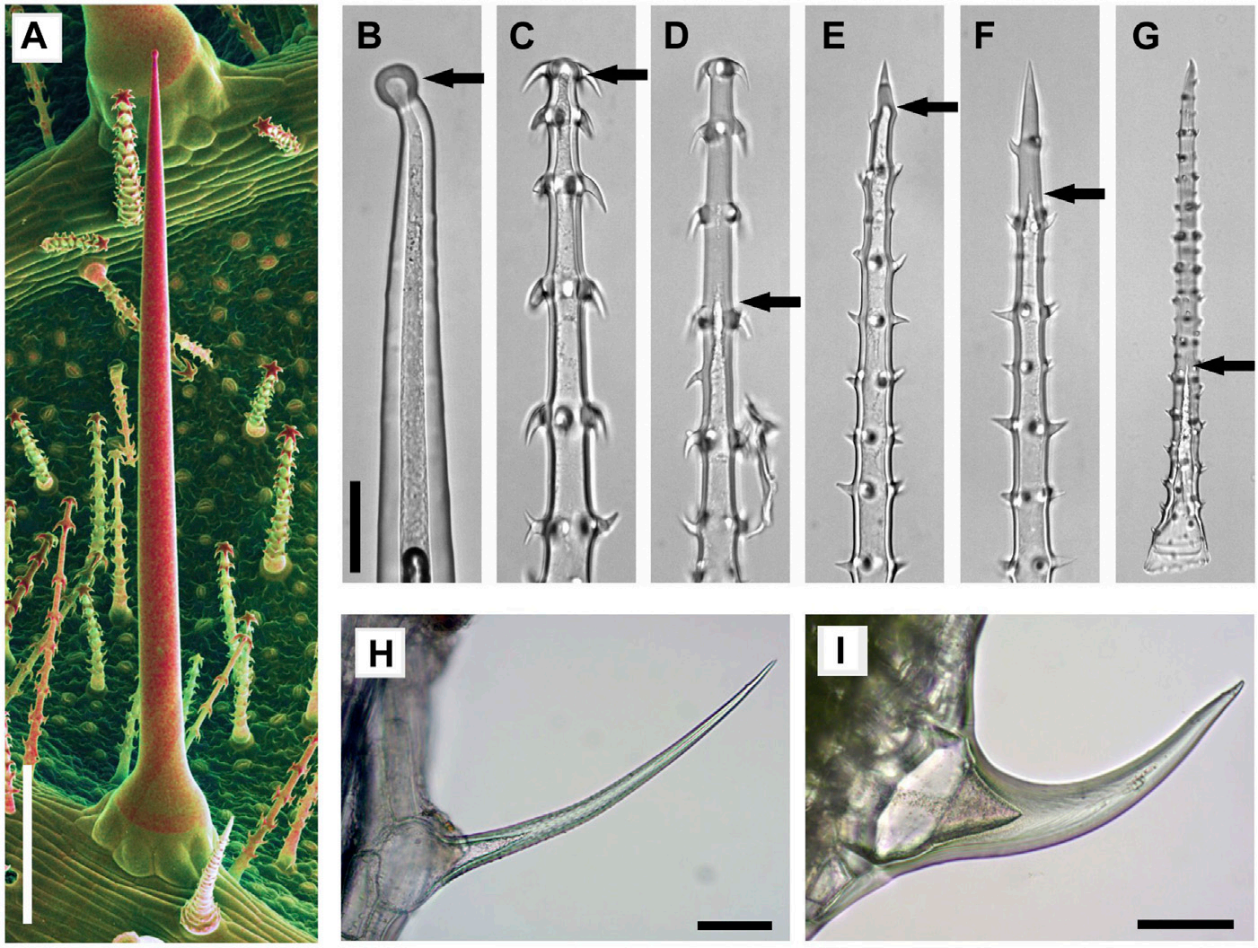
Mineralized trichomes of *Loasa pallida* **(A–G)**, *Urtica dioica* **(H)**, and *Galium aparine* **(I)**. **(A)** SEM image of leaf underside with stinging hair and small glochidiate trichomes. Combined topographic and compositional contrast images showing mineralized structures in red color. Light microscopic (LM) images of a stinging hair **(B)**, glochidiate trichomes **(C–D)** and scabrid trichomes **(E–G)**. Stinging hairs and young stages of scabrid-glochidiate trichomes are hollow; they later become massive by fillings with mineral deposits **(C**, **E**, **F)**. Arrows indicate the end of hollow lumen. Small trichomes of *U*. *dioica* **(H)** and *G*. *aparine* **(I)** which are almost completely massive. Scale bars: A = 500 μm; B-G = 50 μm; H-I = 100 µm.

Biomineralization is a well-known phenomenon in plants and animals (Skinner and Jahren, 2003). In plants, biomineralization is usually found at the level of individual cells, either in epidermal cell walls or as intracellular structures such as cystoliths. Plant biomineralization shows surprising structural and compositional diversity. Calcium oxalate is generally found as intracellular biomineral whereas calcium carbonate is both found as intracellular cystoliths and in the cell walls of trichomes and stinging hairs (Franceschi and Horner, 1980; Lanning and Eleuterius, 1989; He et al., 2014). Silica is the hardest biomineral and widely reported in the form of deposits in the outer cell walls of the epidermis and trichomes, but also as intracellular phytoliths (Nawaz et al., 2019; Gallaher et al., 2020). Abrasive grainy cell wall inclusions and mineralized sharp tips of trichomes, as well as a dense cover of stiff mineralized hairs forming a physical barrier, are assumed to provide protection against grazing animals (Figure 1). The diversity of structural plant biominerals has recently been expanded by the discovery of calcium phosphate-based structures in plants (Ensikat et al., 2016; Ensikat et al., 2017; Weigend et al., 2018). Our SEM studies on the complex plant trichomes of Loasaceae (“Rock nettles,” Cornales) revealed the presence of calcium phosphate in high concentrations at specific sites in the cell wall, such as trichome tips, while the bulk of the trichome wall is typically mineralized with calcium carbonate. Additional studies have demonstrated its wide distribution across a range of plant families including Urticaceae, Boraginaceae, and Brassicaceae since the first discovery of calcium phosphate as a structural plant biomineral in Loasaceae (Mustafa et al., 2018a; Mustafa et al., 2018b; Weigend et al., 2018). Loasaceae are known for morphologically remarkably diverse trichome cover consisting of stinging hairs, much smaller barbed glochidiate trichomes, and scabrid trichomes with sharp tips (Weigend, 2003; Figure 2). Light microscopic (LM) images of Loasaceae trichomes (Figures 2B–G) illustrate the thickness of the walls and whether the trichomes are hollow or massive. Stinging hairs (Figure 2B) are essentially hollow, whereas the small glochidiate and scabrid trichomes are initially hollow, but later become massive (Figures 2C–G). Similar filling of small trichomes occurs in numerous species, such as *Urtica dioica* and *Galium aparine* (Figures 2H,I).

Detailed ontogenetic studies demonstrated that mineralization is initiated at the apex and in the trichome hooks and proceeds towards the trichome base; younger, partially mineralized trichomes still may have a non-mineralized base (Mustafa et al., 2017).

The three biominerals reported in plant trichomes are also found in the animal kingdom. Calcium phosphate and calcium carbonate, and to a lesser degree silica, play a major role in medicine, such as in bone formation. This and other applications are the basis for a rapidly growing body of studies on biomimetic materials based on apatite (calcium phosphate) (e.g., Vandecandelaere et al., 2012; Cazalbou et al., 2015; Haider et al., 2017; Nesseri et al., 2020; Bertolotti et al., 2021), calcium carbonate (Sommerdijk and de With, 2008; Wu and Wang 2013) and silica (Knecht and Wright, 2003; Jackson et al., 2015; Piletska et al., 2017). Despite this broad range of studies, very few focus on the exact localization, formation, composition and properties of plant biominerals. Trichome biomineralization is particularly useful for functional and physiological studies because the process of biomineralization can be studied more or less directly with suitable techniques. Previous studies examined the trichome surfaces for their elemental composition with various SEM-techniques, but we have little knowledge of the chemical composition of the cell walls below the surface, even though this likely plays a major role in functionality. Transmission electron microscopic (TEM) studies of thin sections and light microscopy of stained sections revealed the stratification of silicified and calcified cell wall structures of stinging hairs, and the different composition of stinging hair walls in Loasaceae, Euphorbiaceae, and Urticaceae have been known for a long time (Thurston, 1969; Thurston and Lersten, 1969), but the analytical capabilities in TEM were very limited at that time. Recently, Hughes et al. (2017) studied mineral deposits and stratification of biominerals in *Urtica* stinging hairs, while recently published detailed analyses of element distribution in stinging hairs, particularly of the genus *Nasa*, demonstrate complex deposition patterns of silica and calcium phosphate in trichome apices (Mustafa et al., 2017; Mustafa et al., 2018b). Raman spectroscopy as an analytical method with light microscopy resolution provides compositional data about organic and inorganic components of the samples with the possibility of analysing living trichomes, if they are kept immersed in water to avoid heat problems (Ensikat and Weigend, 2019).

We focus on species with triple biomineralization, i.e., containing SiO_2_, CaCO_3_, and Ca-phosphate (Ensikat et al., 2017) including the differential distribution of organic substances. Patterns of biomineralization at the micro-scale are investigated by sectioning and SEM block-face imaging of embedded samples. Complementary data from Raman spectroscopy are provided to understand both the inorganic compounds in trichome walls and their associated organic matrix substances. Our study strives to advance our knowledge of plant biomineralization and provide the basis for further exploration with sophisticated and high-resolution techniques and the tools of molecular physiology.

## Materials and Methods

### Plant Material

For the study of trichome mineralization we used species of three Loasaceae genera (*Blumenbachia*, *Caiophora*, *Loasa*) that are known for complex biomineralization, and for the purposes of comparison, *Urtica dioica*. All plants used in this work were cultivated in the Botanical Gardens of the University Bonn, Germany. The following species appear in this study: *Blumenbachia insignis* (accession 36150, Herbarium T. Jossberger 1210); *Caiophora clavata* (accession 33612, T. Jossberger 548); *Loasa pallida* (accession 36565, T. Jossberger 666). Fully developed leaves of adult plants were used; however, mineralization patterns may vary due to seasonal changes as trichomes undergo distinctly individual development (Mustafa et al., 2017).

### Microscopy

Scanning electron microscopy was performed with a Stereoscan S 200 SEM (Cambridge Instruments, Cambridge, United Kingdom) and a LEO 1450 SEM (Cambridge Instruments), equipped with secondary electron (SE) and backscattered electron (BSE) detectors and an energy-dispersive X-ray (EDX) element analysis system (Oxford Instruments, Oxford, United Kingdom) in conventional high-vacuum mode. Light microscopy was done with a Zeiss Axio Scope (Carl Zeiss GmbH, Oberkochen, Germany).

### Sample Preparation for Scanning Electron Microscopy

Various preparation methods were applied because previous studies had shown that a conventional fixation with formaldehyde solutions could cause artefacts such as the dislocation of calcium compounds, even though silica and calcium phosphate deposits usually appeared unaltered. Alternatives such as freeze substitution for leaf pieces or rapid dehydration of isolated trichomes could avoid such artefacts. Surface images were taken from critical-point (CP)-dried leaf pieces or isolated trichomes. Block-face imaging of resin-embedded samples was used for the analysis of sections. Light microscopy (LM) was performed on isolated trichomes.

### Conventional Ethanol-Formaldehyde Fixation

Leaf pieces of 5–10 mm size were fixed in 70% ethanol +4% formaldehyde for ca. 24 h, dehydrated with ethanol and acetone, followed by critical-point (CP) drying or embedding in “Agar Low viscosity resin” (Plano GmbH, Wetzlar, Germany) for sectioning, according to the product instructions.

### Rapid Dehydration of Isolated Trichomes

Leaves were frozen in liquid nitrogen and the trichomes were scraped off the leaves with a knife blade. Then the trichomes were collected and washed in ethanol and subsequently in acetone. Trichome samples for immediate SEM-imaging were air-dried. For sectioning, trichomes were embedded in resin.

### Freeze-Substitution

Small pieces of fresh leaves were rapidly immersed in cold acetone at its freezing temperature (−95°C), then stored for 3 days at −80°C and further 3 days at −30°C in acetone. After applying fresh acetone at room temperature, the samples were either CP-dried or embedded in resin for sectioning. The use of osmium tetroxide was omitted as osmium would interfere with the EDX detection of elements such as phosphorus and silicon. Prior to SEM imaging, the samples were sputter-coated with a thin layer of palladium (Pd) which, in contrast to gold, does not compromise EDX detection of the elements of interest.

Sectioning was done with an ultra-microtome (Reichert OM U3, Reichert AG, Wien, Austria) using a diamond knife.

### Raman Spectroscopy

For Raman spectroscopy isolated trichomes were carefully washed to remove any remnants of cell plasma, dehydrated with acetone and air-dried. Raman spectra were collected with a confocal Horiba Scientific LabRam HR800 Raman spectrometer (Horiba Europe GmbH, Oberursel, Germany) at the Institute of Geosciences of the University of Bonn, Germany. The Raman spectra were excited with a 2 W frequency-doubled solid state Nd:YAG laser (532 nm); the laser power was adjusted to less than 20 mW at the sample surface. The scattered Raman light was dispersed by a grating with 600 grooves/mm and detected by an electron multiplier charged-coupled device (EM-CCD). A ×50 long-distance objective with a numerical aperture of N.A. = 0.5 was used for all measurements.

### Image Processing

Colorized SEM images were rendered with standard image processing software “Corel Paint Shop Pro X9^©^.” The images from different detectors were combined with the software function “Combine HSL”. In combined SE-BSE images, the SE image was used for the lightness (L) channel, and the BSE signal was used to render a color shift so that mineralized structures appear red. Element mapping images were used to determine color saturation (S) and hue (H), so that the silicon signal causes a color shift towards red and the phosphorus signal shifts the color towards green.

## Results

Scanning electron microscopy (SEM) with integrated energy-dispersive X-ray spectroscopy (EDX) for element analysis is an excellent tool for identification of biominerals. However, surface analyses provide only an incomplete picture when mineralization extends into the volume of the sample. In such cases, sections or transmission views with LM, including Raman spectroscopy, are suitable to comprehensively characterize the sample composition.

### Loasaceae Trichomes

For the present study we investigated three taxa of Loasaceae (*Blumenbachia*, *Caiophora*, *Loasa*) with trichomes, each having quite similar morphologies, but remarkable differences in biomineralization. All three species have three different types of mineralized trichomes (Figure 2): stinging hairs with length of 2–4 mm, which resemble the stinging hairs of Urticaceae (Stinging Nettles) (Figures 2A,B); glochidiate trichomes with a blunt apex and numerous basiscopic barbs and a length of typically 0.1–0.4 mm (Figures 2A,C,D); and scabrid trichomes with sharp tips and a length of 0.5–1 mm (Figures 2E–G). Biomineralization in all trichome types is highly site-specific for all three elements (Si, P, and Ca) examined.

### Three Biominerals in Single-Celled Trichomes

**FIGURE 3:**
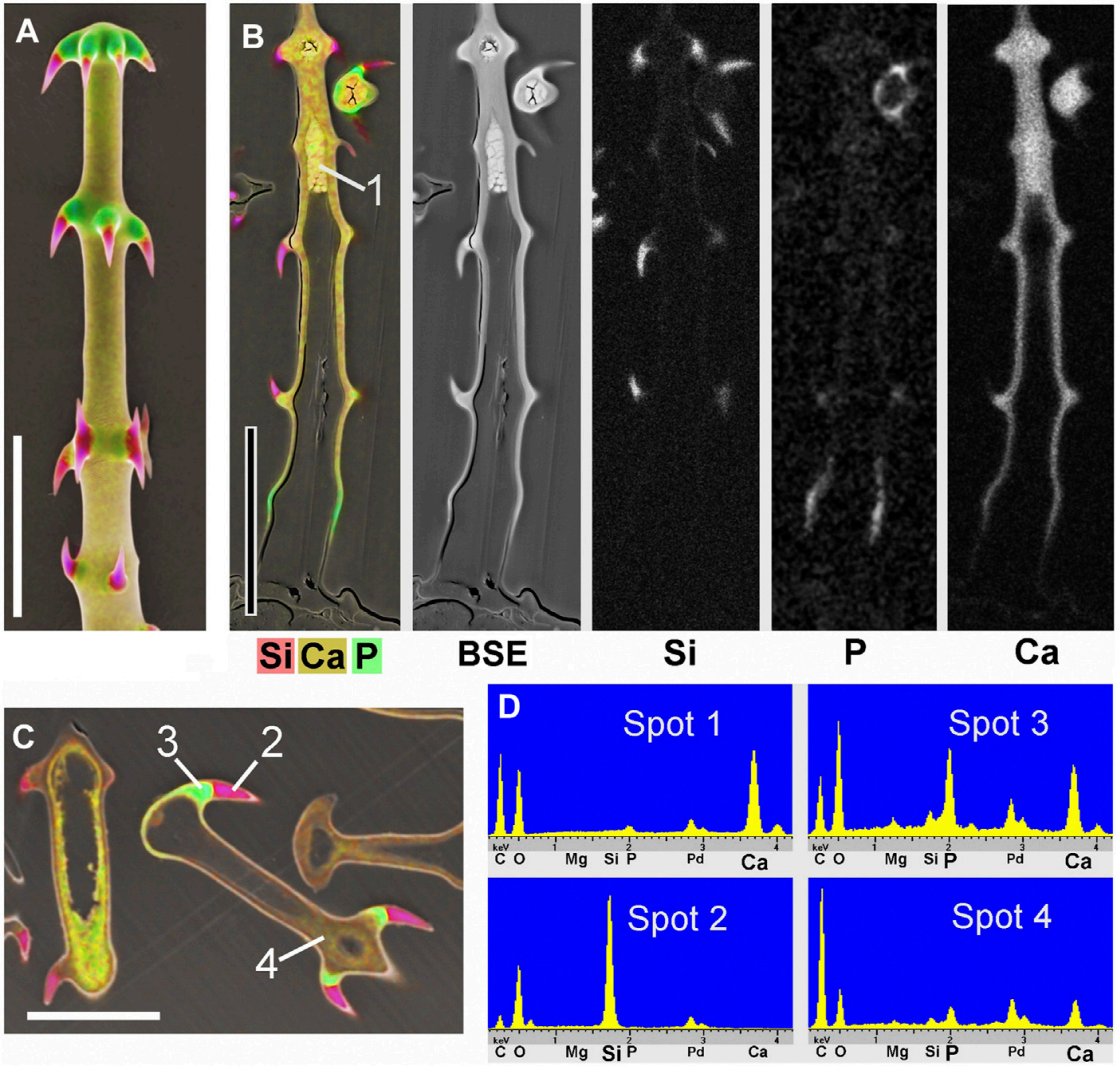
*Caiophora clavata* glochidiate trichomes with three biominerals; element-mapping SEM images and EDX spectra of selected spots. Colours characterize different elements: red = Si; yellow to brown = Ca; green = Ca + P. **(A)** surface view; **(B–C)** longitudinal sections through embedded trichomes treated with different preparation methods: **(B)** Freeze substitution; grayscale mapping images of elements show lower concentrations. **(C)** conventional formaldehyde fixation. **(D)** EDX spectra from four locations indicated in **(B)** and **(C)** indicate different biominerals: calcium carbonate (Spot 1); silica (Spot 2); calcium phosphate (Spot 3); but only low Ca concentration in cell lumen after formaldehyde fixation (Spot 4). The freeze substitution-prepared sample **(B)** shows well-preserved Ca-minerals in cell wall and lumen, silica in barbs, and P in basal cell wall. Conventional preparation **(C)** caused irregular deposits in cell lumen and loss of Ca in walls, but silica and calcium phosphate structures in barbs appeared well preserved. Scale bars: A-B = 50 μm; C = 30 µm.

Figure 3 shows images of glochidiate trichomes of *Caiophora clavata*. The coloured SEM images combine topography and element-mapping images, to highlight the elements in different colours: Si in red, Ca in yellow to brown, P (together with Ca) in green. SEM surface images show the composition of the outermost layer of the cell wall close to the surface, depending on the penetration depth of the electron beam (typically a few micrometers). Figure 3B shows longitudinal sections in combined colour images and separate grayscale mapping images for each mineral element; a surface image of a similar trichome is shown for comparison (Figure 3A). A detailed image of the barbs (Figure 3C) shows Si in the barb tips and strong signals of P and Ca in the barb shoulders. A comparison of Figures 3B,C illustrates differences in quality between two different preparation methods. The sample in Figure 3B was prepared by “freeze substitution,” which preserves the calcium carbonate-based minerals particularly well. Location “1” shows the Ca-rich core in the cell lumen, which appears well preserved, as well as the calcium content of the cell wall, in contrast to the conventionally prepared trichome in Figure 3C with irregular deposits in the cell lumen and a loss of minerals in the walls. Silica in the barb tips and calcium phosphate in barb shoulders appear well preserved in both samples. Ca-phosphate is also found in the base of the trichome in Figure 3B. EDX spectra of selected spots (Figure 3D) indicate high concentrations of calcium carbonate (Spot 1), silica (Spot 2), calcium phosphate (Spot 3), and only low concentrations of Ca and P in the form of an irregular precipitation in Spot 4.

### Silica in Barbs and in the Entire Shaft Wall

**FIGURE 4:**
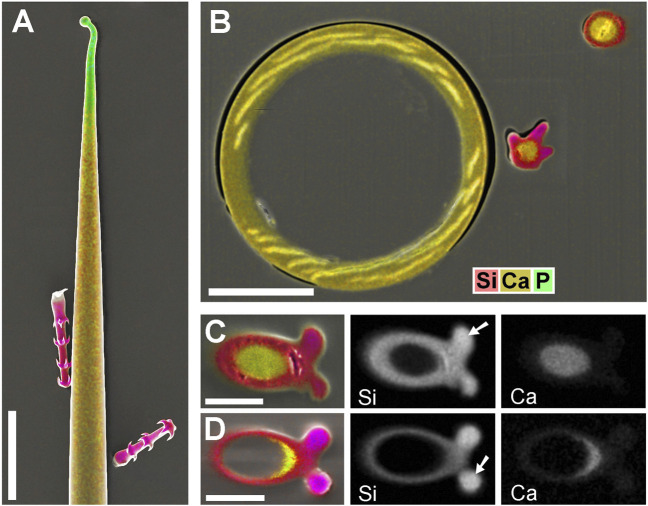
*Blumenbachia insignis*, combined topographic and element-mapping images of stinging hair and glochidiate trichomes. Colors: Si shown in red, Ca in yellow, and P in green. **(A)** Surface view of part of a stinging hair (yellow, green) and two glochidiate trichomes (red, indicating Si). **(B)** Section through a similar arrangement of trichomes shows that stinging hair wall is mineralized with Ca compounds across entire thickness. Glochidiate trichomes have a silicified regular wall, very high Si concentration in barbs (arrows), and Ca in deposits in cell lumen. **(C–D)** Cross sections through massive and hollow regions of glochidiate trichomes including barbs; combined colour images and separate element maps of the Si and Ca distribution. Scale bars: A = 100 μm; B = 30 μm; C-D = 10 µm.

*Blumenbachia insignis* has a unique mineralization pattern (Ensikat et al., 2017): the cell walls of glochidiate trichomes are entirely silicified whereas the stinging hairs contain calcium compounds only. Figure 4 A is a surface view; Figure 4B shows a cross section through a similar assembly of glochidiate trichomes and a shaft of a stinging hair. The stinging hair wall is homogeneously mineralized with calcium compounds (presumably carbonate, yellow). Cell walls of glochidiate trichomes are mineralized with silica, including the barbs. The lumen of massive trichomes is filled with calcium carbonate. Sections through a massive and a hollow glochidiate trichome are shown in Figures 4C,D. The silicon distribution map shows the highest Si concentration in the barbs (arrows).

### Calcium Phosphate/Calcium Carbonate Distribution

**FIGURE 5:**
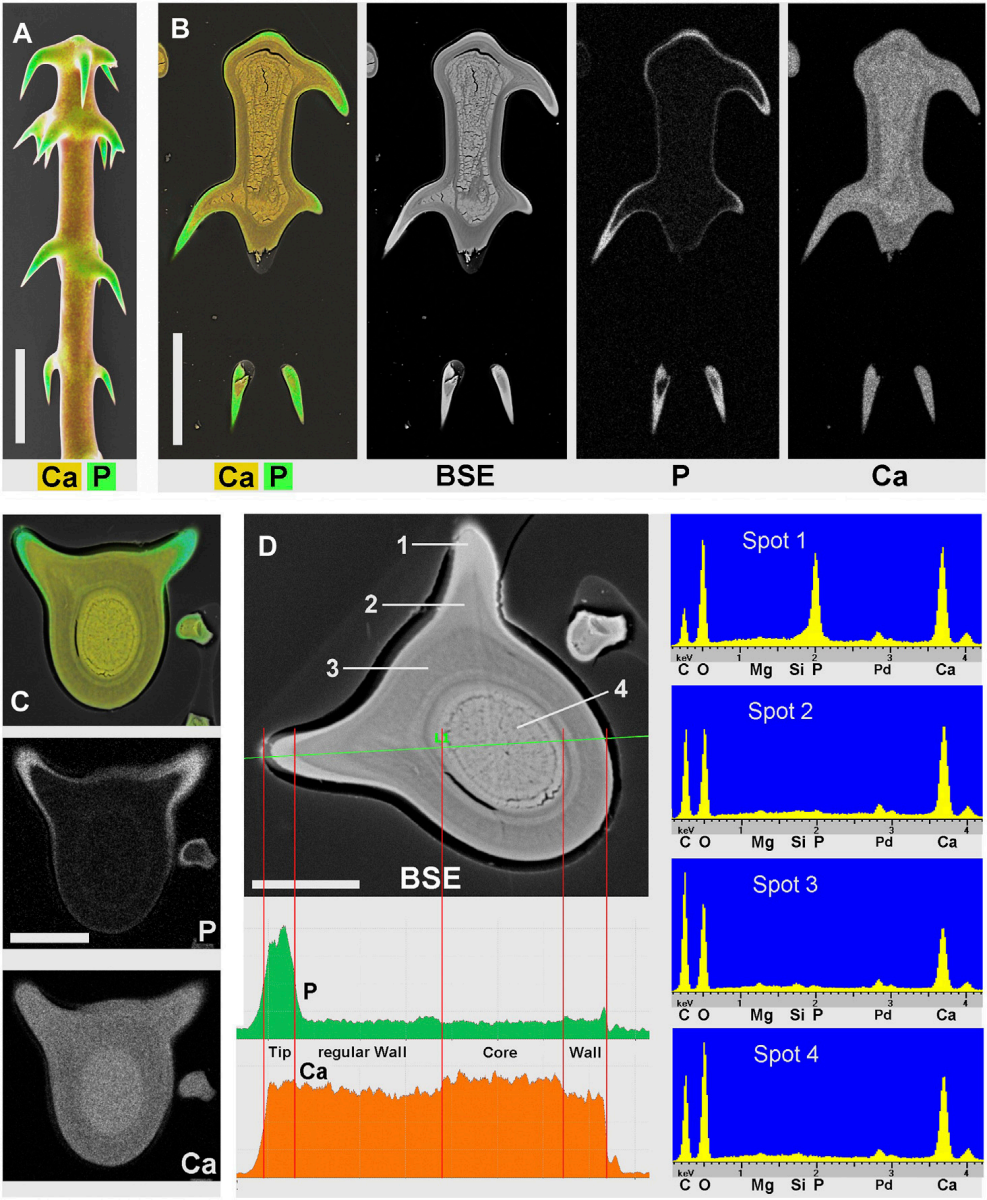
*Loasa pallida*; element-mapping images and analyses of glochidiate trichomes. Colors indicate Ca in yellow to brown, P in green (together with Ca). **(A)** surface view; **(B)** longitudinal section through upper part of an embedded trichome. BSE image illustrates total mineral concentration with better resolution than EDX element mapping. P is found in the barb tips, as an apical cap, and as a thin surface coating, Ca concentrations are highest in barbs and inner deposits and slightly lower in the wall of the shaft. **(C–D)** Analysis of a single trichome cross section shows Ca and P distribution in the element-mapping images **(C)**, whereas a BSE compositional contrast image **(D)** shows different mineral concentrations and stratification due to higher resolution. EDX spectra of selected spots and a line-scan show different Ca concentrations quantitatively. (Spots: 1 = barb tip; 2 = barb shoulder; 3 = regular wall; 4 = core.) Note the low carbon concentration in spectrum of Spot 1 from calcium phosphate-rich barb tip. Scale bars: A-B = 50 μm; C-D = 20 µm.

Most *Loasa* species lack silica in their trichomes. Instead, stinging hair tips and glochidiate trichome barbs are mineralized with calcium phosphate. Figure 5A,B show the distal part of a glochidiate trichome of *Loasa pallida* in surface view and as longitudinal section. Element mapping images show the distribution of P and Ca. The barb tips and a thin layer on the apical cap contain calcium phosphate (P and Ca). The lumen is filled with calcium; the higher brightness indicates a slightly higher Ca concentration than in the wall of the shaft, which is also mineralized with calcium. A cross section of a glochidiate trichome (Figures 5C,D) was analysed by element mapping, spot spectra of selected locations, a BSE image, and an EDX line-scan that shows a concentration profile. The phosphorus map (Figure 5C) shows high concentrations in the barbs and a thin outer layer. The BSE image (Figure 5D) shows this phosphate layer with better resolution as a separate zone sharply delimited from the calcified wall. The EDX spectrum of this region (Spot 1) shows high P-to-Ca ratios and low carbon concentrations. The barb base (Spot 2) and the cell wall (Spot 3) contain Ca, but almost no P. The lumen filling (core, Spot 4) has a high Ca concentration. The Ca concentration in the wall is slightly lower than in the barb shoulder and core.

**FIGURE 6:**
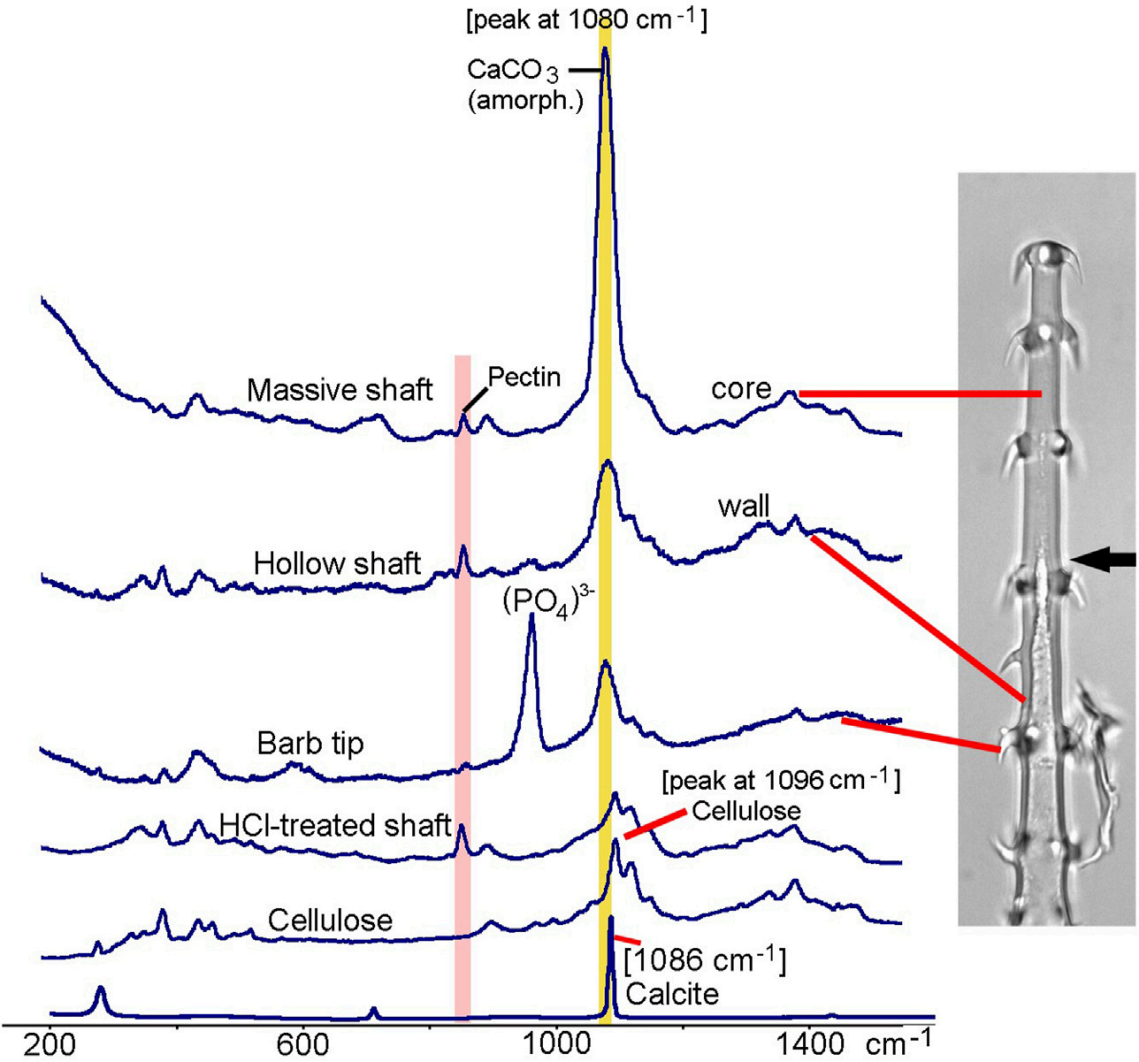
*Loasa pallida*, Raman spectroscopy analyses of glochidiate trichomes. Raman spectra of core of massive shaft, wall of hollow shaft, a barb tip, and for comparison spectra from a demineralized, HCl-treated trichome and from cellulose and calcite. The broad carbonate peak at 1080 cm^−1^, which overlaps with the main Raman band of cellulose at 1096 cm^−1^, indicates amorphous or disordered calcium carbonate, with high concentration particularly in the core. A peak at 856 cm^−1^ indicates Pectin which is present in trichome walls even after HCl treatment. Barb tips show an intense phosphate peak at 958 cm^−1^ and smaller carbonate peak at 1080 cm^−1^. HCl-treated trichomes show Raman bands typical for carbohydrates such as cellulose and pectin.

In addition to EDX, we used Raman spectroscopy to further characterize the minerals and the organic components of trichome walls. Figure 6 shows Raman spectra from the massive and hollow regions of a glochidiate trichome, from a barb tip, and for comparison the spectra of a demineralized (HCl-treated) trichome and of cellulose and crystalline calcium carbonate (calcite). A strong carbonate peak can be identified in the massive, distal portion of the trichome; the hollow trichome wall shows a much smaller carbonate peak. The width of the carbonate band and the peak position at 1080 cm^−1^ are indications for amorphous calcium carbonate, whereas crystalline calcite is characterised by a sharp Raman peak at 1086 cm^−1^. Most of the other minor peaks can be assigned to cellulose, except for the 856 cm^−1^ pectin peak (Chylinska et al., 2014). The barb tip spectrum is dominated by a phosphate peak which indicates calcium phosphate, and a smaller carbonate peak at 1080 cm^−1^.

**FIGURE 7:**
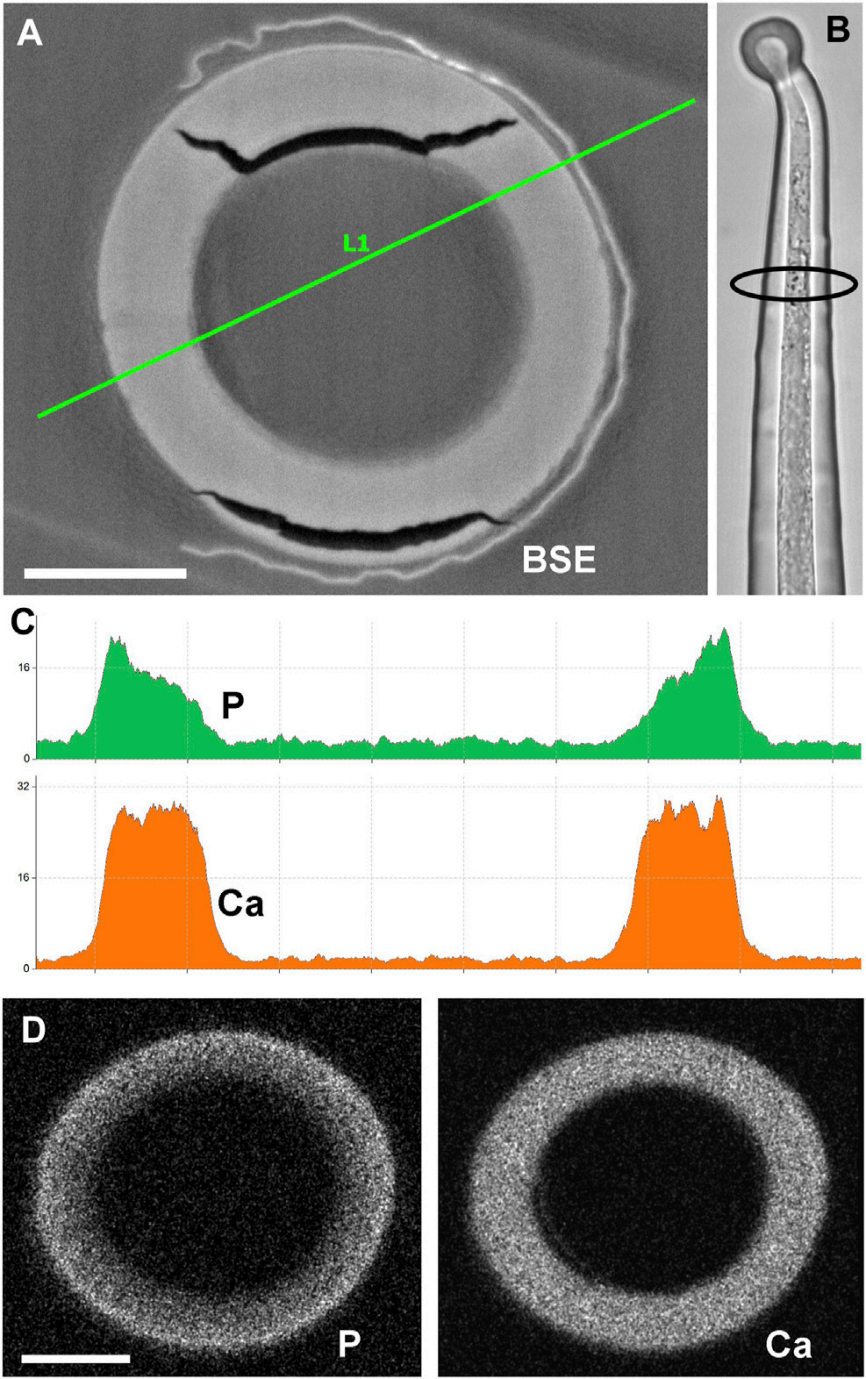
Cross section of embedded stinging hair of *Loasa pallida* and analysis of P and Ca distribution. **(A)** BSE image with EDX linescan **(C)**; the LM image **(B)** illustrates the position of the section close to the apex. The line-scan concentration profiles and element mapping images **(D)** across the cell walls show a continuous decrease in P concentration from outside to inside, contrasting with a constant Ca concentration across the wall. Scale bars: 10 µm.

The stratification of different calcium phosphate and carbonate phases shown in Figure 4 is in remarkable contrast to previous analyses of stinging hair sections of *Loasa pallida*, where we had found a continuous change in the phosphorus concentration from the inner to the outer side of the wall (Ensikat et al., 2016). Thus, we prepared new cross sections of stinging hairs in order to check the previous findings (Figure 7). Indeed, the phosphate concentration decreases gradually from the outside of the wall towards the inside of the wall. No separate layers of calcium phosphate and calcium carbonate are formed; calcium phosphate on the outside grades into calcium carbonate on the inside.

**FIGURE 8:**
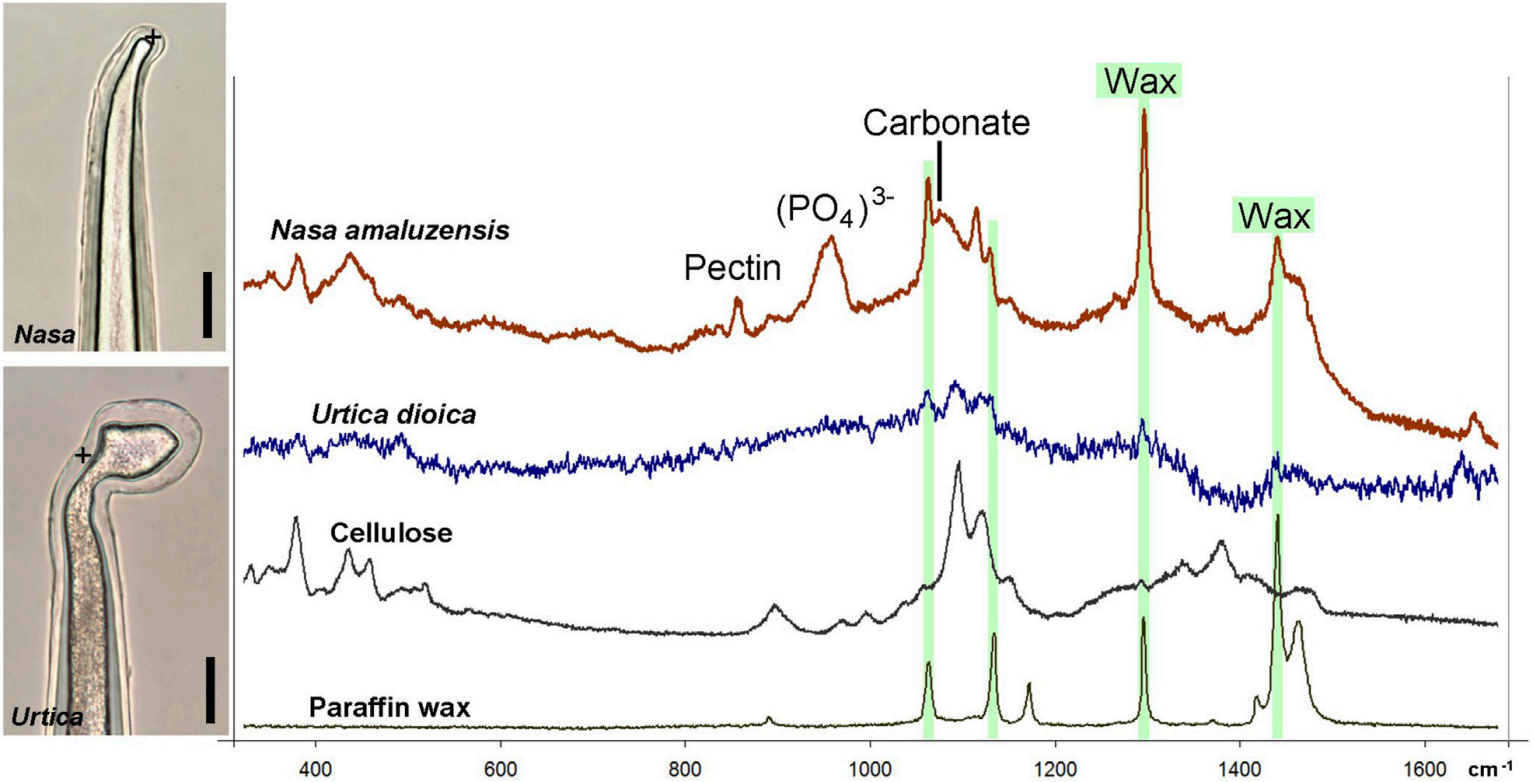
Raman spectra of apices of living stinging hairs of *Nasa amaluzensis* and *Urtica dioica*. Spectra of cellulose and paraffin wax are shown for comparison. Leaf stalks with stinging hairs were immersed in water. Spectrum for the *Nasa* stinging hair tip clearly shows Raman bands of cellulose, pectin, phosphate, and a small carbonate peak which overlaps with the cellulose peak. Several sharp peaks indicate crystalline wax (highlighted in green). The spectrum of *Urtica* shows only traces of cellulose and wax bands. Scale bars: 10 µm.

Raman spectroscopy enables the analysis and detection of organic and inorganic components in trichomes walls in living plants, requiring immersion in water to avoid heat damages. We acquired Raman spectra from the tips of very young stinging hairs of *Nasa amaluzensis* in the process of developing a calcium phosphate tip, and *Urtica dioica* with a silicified tip (Figure 8). The spectrum of *Nasa* clearly shows the Raman bands of cellulose, pectin, phosphate, and a small carbonate peak. Several sharp peaks indicated crystalline wax components, which occur commonly on young, untreated plant surfaces. In contrast, the spectrum of *Urtica* showed only traces of the wax and cellulose peaks; amorphous silica produces no detectable peaks in this region.

### Solubility of Calcium-Based Biominerals in Water

**FIGURE 9:**
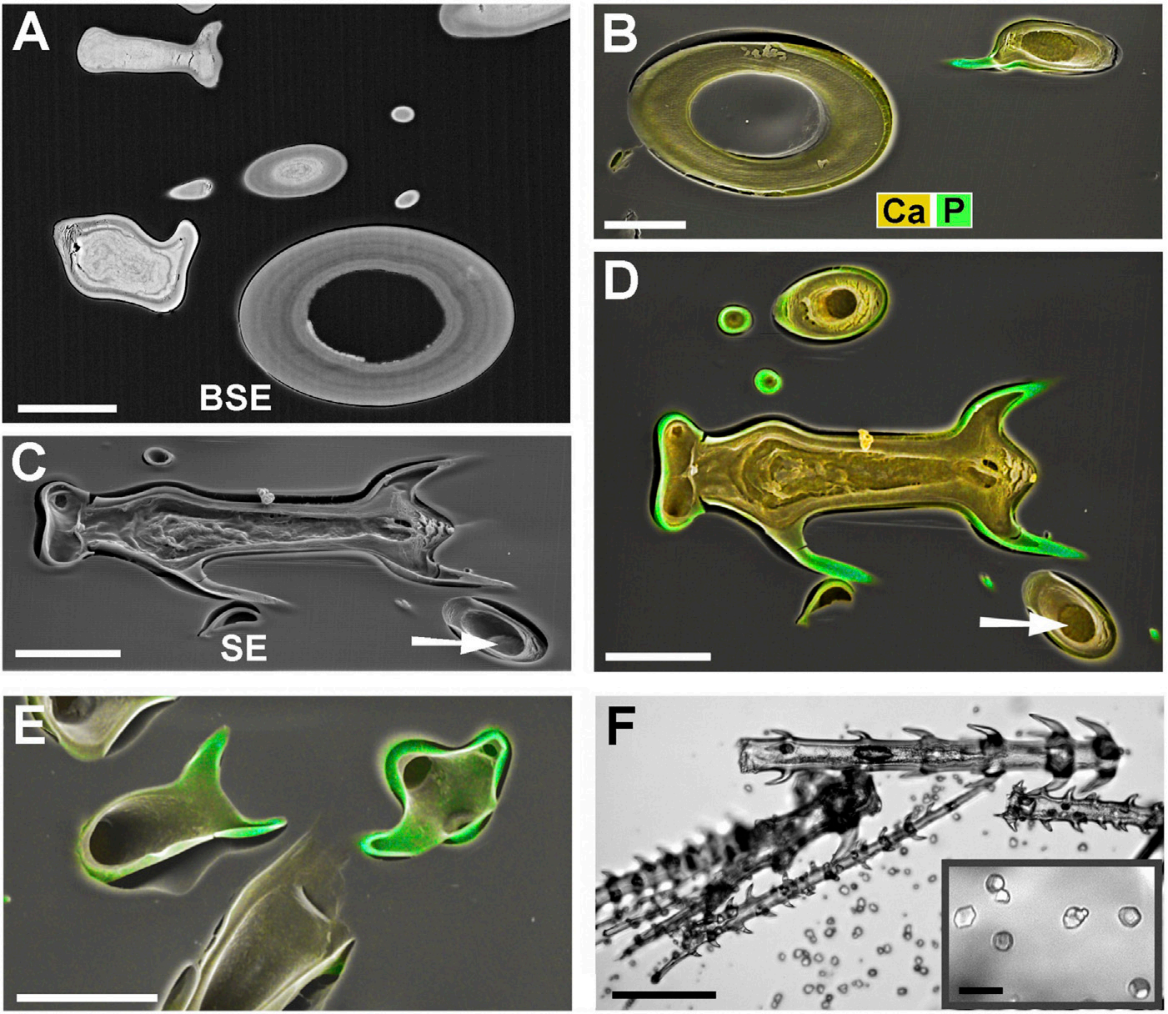
SEM block-face images and LM image of *Loasa pallida* trichomes illustrating solubility of biominerals in water. **(A)** BSE image of sections through glochidiate trichomes and a stinging hair without H_2_O treatment (control). High mineral concentrations appear bright. **(B–E)** Combined SE and element-mapping images of embedded trichomes after 12 min in water (at 29°C) **(B–D)**, and after 2 h in water **(E)**. **(B–D)** After 12 min H_2_O treatment, Ca-carbonate of the core is dissolved to a depth of ca 10 µm. Remaining Ca-carbonate can be seen at the bottom of the holes (arrow). Ca-phosphate in the barb tips is still present (green). Cell walls still contain Ca (yellow) and show slight shrinkage. **(C)** SE image of tilted sample provides a better impression of depth of voids after mineral dissolution. **(E)** After 2 h of H_2_O treatment, the calcium carbonate core has been dissolved completely. The regular cell walls still exist, but have lost most of their mineral content; Calcium phosphate is still present in barb tips and adjacent outer layers (green). **(F)** LM image of a batch of isolated glochidiate trichomes in water after 45 min of immersion. The amorphous CaCO_3_ in the trichomes dissolves and calcite crystals start growing in the vicinity of dense trichome batches. The inset shows growing crystals in detail. Scale bars: A-E = 30 μm; *F* = 100 μm; inset in *F* = 20 µm.

Figure 9 shows blockface images of sectioned embedded trichomes of *Loasa pallida* treated with water (H_2_O) for different intervals demonstrating a striking progression of demineralization: Trichomes without water treatment (A) show massive mineralization mainly with calcium carbonate, both in the cell walls and in the core. After 12 min of water treatment (B-D), the filling of amorphous calcium carbonate in the lumen is largely dissolved up to a depth of approximately 10 µm (arrows); the walls still contain some Ca, indicated by the yellow colour (Ca) or green color (Ca + P). After 2 h of water treatment (E), the trichome is largely demineralized, only the calcium phosphate in the barbs appears to be largely untouched. Figure 9F illustrates the process of calcium carbonate mobilization and precipitation in a batch of isolated trichomes of *Loasa pallida* in water under a light microscope. Calcium carbonate dissolves from the cell lumen and cell walls, forming calcite crystals in the vicinity of the trichomes.

## Discussion

Loasaceae trichomes have previously been shown to display complex patterns of biomineralization (Ensikat et al., 2017). The present study is an extension of these studies to provide a three-dimensional view of biomineralization and a first look at the organic phase in inorganic/organic composites within trichome cells. The most striking result is the extreme zonation of mineral deposition across the cell walls and wall protuberances (barbs), with different minerals deposited with pinpoint precision. Similar complexity of cell wall structures are known from non-mineralized plant tissues. Plant cell walls are, unlike membranes of animal tissues, prominent structures contributing to the mechanical stability. Different cell wall composition, including hydrophilic carbohydrates (mainly cellulose) and hydrophobic components such as lignin, cutin and waxes, determine the permeability for water and ions. Tracheids or the cells of the Casparian strip are two examples, where the functional divergence in different wall portions is mediated by different types and quantities of organic compounds. Our observation of differentially mineralized trichome walls expands this phenomenon to the outer plant surface, where chemical heterogeneity at the micrometre level is expressed with the presence of up to three different mineral components in combination with different organic compounds.

The mineralization patterns in plant trichomes appear to reflect a range of functions, including plant defence. The tips and hooks of the trichomes are rendered particularly hard and sharp by the inclusion of either silica or calcium phosphate, enabling them to damage skin, mucous membranes and/or the exoskeleton of insects and other herbivores. Conversely, stinging hairs retain a certain degree of brittleness, so that the apex can break off and the caustic stinging substances injected into the attacker from the hollow interior of the mineralized trichome cell, functioning as hypodermic syringe (Thurston and Lersten, 1969; Tuberville et al., 1996). In trichomes containing both calcium carbonate and calcium phosphate there tends to be a trend towards phosphate “coatings,” i.e., the part of the trichome exposed to the elements is “varnished” with calcium phosphate, possibly to make use of its lower water solubility, as shown by the leaching experiments. Defensive trichomes in other plant species may be functionally very similar to those of Loasaceae without being mineralized: Stinging hairs of Cnidoscolus are morphologically very similar to those of Loasaceae or Urticaceae, but are entirely made up of organic polymers (Mustafa et al., 2018b). Overall, biomineralization of the cell wall shows extraordinary diversity: Different parts of the cell wall are incrusted with different mineral components (calcium carbonate, calcium phosphate, silica), in different structural contexts and relative concentrations, with varying amounts of an organic matrix. The distal and apical parts, where “rigidity” is of paramount importance, are nearly completely mineralized with minute organic admixtures. Other parts of the trichome, especially the shaft, have a higher proportion of cellulose and/or pectin, as shown by Raman spectroscopy, likely creating a composite material with higher strength and flexibility. In addition to the biomineralization of wall structures, we also find an amorphous “filling” of calcium carbonate in glochidiate trichomes, progressing basiscopically with trichome age. Both the amorphous filling and the calcium carbonate mineralization of the cell walls may be easily mobilized upon contact with water. Solubility of calcium-based biominerals in water has long been known as a problem in preparations for TEM investigations. It has been reported that during the ultramicrotomy of bone tissue sections floating on a water surface lose part of their mineral content; dry sectioning is required to avoid this artefact (Boothroyd, 1964; Thorogood and Gray, 1975; Studer et al., 2011).

Crystallinity is not necessarily an attribute of biominerals. Biogenic silica, for example, is generally amorphous. Biogenic plant calcium carbonate has been found as crystalline calcite in the seed coat (pericarp) of Lithospermum (Hilger et al., 1993; own data, not shown), whereas analyses of trichomes by Raman spectroscopy and X-ray powder diffraction always indicates amorphous material. Admixtures of various additives, such as phosphoproteins or inorganic phosphate, may stabilise amorphous CaCO_3_ (Bentov et al., 2010; Gal et al., 2010).

The biomineral silica widely occurs as nearly pure substance in very hard structures or surface coatings. Thus, its integration into the wall of glochidiate trichome shafts of *Blumenbachia* is a remarkable novelty. In several species of other plant families, e.g., *Urtica mairei* and *Urera baccifera*, cell walls of trichomes are mainly mineralized with calcium phosphate instead of carbonate (Mustafa et al., 2018b). Thus, all three biominerals calcium carbonate, calcium phosphate, and silica can occur in concentrated form (in trichome tips or fillings in the cell lumen) or as composites with cellulose and pectin in the cell walls. Biomineralization in plants, particularly the formation of calcium carbonate and phosphate, has received little attention in the past, even though calcium phosphate biomineralization is of considerable interest in medicine and for comparatives studies of the process of biomineralization in animals such as vertebrates (Dorozhkin and Epple, 2002; Dillon et al., 2019). Exploration of the physicochemical properties of biominerals is a promising field of research for biomedical applications (Al-Kattan et al., 2012; Credou and Berthelot, 2015; Haider et al., 2017; Konovalova et al., 2017; Markov et al., 2017; Hickey and Pelling, 2019; Nesseri et al., 2020; Tschon et al., 2020) and may get a novel stimulus by the expansion into carbohydrate-based biomineral composites.

Recent research focusses on the chemical and physical properties of plant biominerals and the physiological pathways leading to their formation (Kumar et al., 2021). The accessibility of plant trichomes for *in vivo* studies and analysis by light microscopy and Raman spectroscopy renders them highly suitable subjects for such studies.

## Data Availability

The original contributions presented in the study are included in the article/Supplementary Material, further inquiries can be directed to the corresponding author.
